# Stem cells out of the bag: characterization of *ex vivo* expanded mesenchymal stromal cells for possible clinical use

**DOI:** 10.2144/fsoa-2019-0129

**Published:** 2020-01-06

**Authors:** Sérgio M Lopes, Susana Roncon, Filipa Bordalo, Fátima Amado, Sara Ferreira, Ana C Pinho, Joana Vieira, Altamiro Costa-Pereira

**Affiliations:** 1 Cellular Therapy Department, Instituto Português de Oncologia do Porto, Porto, Portugal; 2 Genetics Department, Instituto Português de Oncologia do Porto, Porto, Portugal; 3 Center for Health Technology & Services Research (CINTESIS), Faculty of Medicine, University of Porto, Porto, Portugal; Department of Community Medicine, Information & Health Decision Sciences (MEDCIDS), Faculty of Medicine, University of Porto, Porto, Portugal

**Keywords:** adipogenic differentiation, bone marrow, collection bag, filter system, mesenchymal stromal cells, mixed lymphocyte reaction, mononuclear cells, MSC

## Abstract

**Aim:**

Mesenchymal stromal cells (MSC) are a promising tool for cellular therapy and regenerative medicine. One major difficulty in establishing a MSC expansion protocol is the large volume of bone marrow (BM) required. We studied whether cells trapped within a collection bag and filter system could be considered as a source of MSC.

**Results:**

From the 20 BM collection bag and filter systems, we recovered an average of 1.68 × 10^8^ mononuclear cells, which is the equivalent to 60 ml of filtered BM. Mononuclear cells were expanded *ex vivo* to 17 × 10^6^ MSC, with purity shown by a CD44^+^, CD105^+^, CD90^+^ and CD73^+^ immunophenotype, a reduction of 20% proliferating cells in a mixed lymphocyte reaction and also the ability of adipocyte differentiation.

**Conclusion:**

Long-term MSC cultures were established from the usually discarded BM collection bag and filter, maintaining an appropriate phenotype and function, being suitable for both investigation and clinical settings.

## Lay abstract

Mesenchymal stromal cells (MSC) are a promising tool for cellular therapy and regenerative medicine. One major difficulty in obtaining MSC is the large volume of bone marrow (BM) required from a healthy donor. From usually discarded collection bags of BM collected for transplant, we recovered a number of cells equivalent to 60 ml of BM and expanded functional MSC with high purity. We believe that those recovered cells are an alternative to BM for obtaining MSC. The routinely recovery of such cells in reference centers, in a way similar to a public cord-blood bank, could benefit the scientific community, once further research is conducted to confirm results.

Bone marrow (BM) is the primary hematopoietic tissue in an adult. First recognized as the home of hematopoietic progenitor cells (HPC), it also contains progenitor cells for other lineages [1]. Although the use of BM in the hematopoietic stem cell transplant (HSCT) setting has declined in the last decades, its interest in cellular therapy is increasing, with numerous protocols using BM-derived mononuclear cells (MNC) as a primary source, such as *ex vivo* expansion of HPC and mesenchymal stromal cells (MSC) differentiation.

MSC present some unique features, namely the ability to modulate the immune system, which can be applied in hampering both alloreactivity, such as post-HCT graft versus host disease (GvHD), autoreactivity, such as autoimmune disorders and also in chronic inflammation [2,3]. Although less investigated than in GvHD, there also seems to be a future for MSC in preventing rejection in solid organ transplants [4,5].

Furthermore, MSC can escape T-cell recognition and exert immunomodulation in an HLA-independent manner, making every MSC donor a potential universal donor.

Another attractive feature of MSC is their ability to differentiate into any tissue of the mesoderm lineage, such as bone, cartilage and adipose tissue. This is an extremely powerful tool in the field of regenerative medicine. MSC are almost ubiquitous in the body and can be isolated from a vast array of tissues, most frequently from the BM and adipose tissue [6].

MSCs have been shown to interact with HPC by controlling or directly providing a stem cell niche for HSCs, with the ablation of MSC resulting in disrupted hematopoiesis [7,8]. Under the appropriate experimental conditions, MSC can even be used to obtain HPC [9]. The therapeutic potential of the MSC–HPC interaction in restoring the stem cell niche can also be explored in the context of severe aplastic anemia, where the co-infusion of MSC seems to reduce graft failure [10].

Since the first demonstration that MSC can be safely expanded *ex vivo* and then reinfused [11], many studies have been published using different sources, expansion protocols and target populations, confirming the safety of this procedure [12]. These combined features give MSC a great ‘off the shelf’ potential [13], as opposed to other cellular therapy products, which have to be tailor-made for each individual patient.

One major difficulty in establishing an MSC expansion protocol, especially in an investigative setting, is the access to healthy donors and the volume of BM required.

The standard BM collection protocol for HSCT requires multiple aspirations from the iliac crests, which are pooled in a collection bag containing an anticoagulation solution (ACD-A). Prior to infusion to the patient or further manipulation, the BM needs to be filtered in order to remove fat, cell clumps and bone fragments. This filtering process is performed by connecting the collection bag containing the BM to a 200 μm mesh filter, which, in turn, is connected to a new bag, under sterile conditions. Although the co-infusion of cells recovered from washing the collection bag and filter system (bag/filter) has been described as beneficial to the patient [14], in most centers, ours included, the bag/filter is considered clinical waste and is discarded.

In this study, we aim to evaluate whether MNC can also be isolated from the BM collection bag/filter and expanded *ex vivo* into functional MSC, with potential for post-transplant cellular therapy.

## Materials & methods

### MNC isolation from collection bags

Bone marrow samples were harvested from 20 healthy adult donors intended for allogeneic HSCT, both related and unrelated. Donor information is summarized in Table 1. After filtering and distribution was complete, the collection bag/filter were anonymized and transported to the lab to be processed.

**Table 1.** Mononuclear cell recovery and viability for each donor.

Sample	Age	Sex	Recovered MNC × 10^6^		Estimated BM volume (ml)	Recovered MNC viability (%)
			Bag/filter	Filtered BM sample		
BM1	37	F	43.9	1.4	31	94
BM2	31	F	201.3	4.5	44	96
BM3	32	F	103.3	1.5	69	93
BM4	24	F	662	3.7	18	97
BM5	41	M	78.6	1.2	68	96
BM6	33	F	238.5	15.4	39	95
BM7	28	F	119.2	1.6	76	98
BM8	42	F	180.0	2.8	65	96
BM9	43	F	66.3	1.1	61	94
BM10	31	F	123.4	1.4	87	95
BM11	23	M	140.9	2.4	58	93
BM12	27	M	215.7	3.0	71	92
BM13	42	M	55.4	2.2	25	99
BM14	25	F	61.4	0.8	58	96
BM15	22	M	181.5	11.7	12	94
BM16	45	M	54.4	4.4	15	95
BM17	48	F	165.5	4.5	48	92
BM18	35	M	249.0	1.9	49	96
BM19	51	M	645.2	4.2	214	94
BM20	26	F	373.0	4.7	84	93

Comparison of number of mononuclear cell isolated from the filtered bone marrow sample with the correspondent bag/filter washing.

BM: Bone marrow; MNC: Mononuclear cell.

The collection bag/filters were washed and rinsed under aseptic conditions: 30 ml of RPMI 1640 (Thermo Fisher Scientific, MA, USA) were added to the bag, stirred and passed through the original filter and the procedure repeated with fresh RPMI 1640; 30 ml of cell suspension was slowly layered on 20 ml of Lymphoprep™ (Axis-Shield, Dundee, UK) in 50 ml conical tubes and centrifuged for 20 min at 1600 r.p.m. The MNC fraction was carefully aspirated with a pipette into a new 50 ml tube, topped with RPMI 1640 and centrifuged for 10 min at 1100 r.p.m; supernatant was discarded and the cells were resuspended in FACSFlow (BD Biosciences, NJ, USA) for viability assessment and enumeration. From every BM processed at our center, 1 ml of filtered BM was taken to assess the clonogenic capacity as part of standard product quality evaluation. This procedure requires an initial step of MNC isolation by the same technique, to determine how many MNC can be isolated per milliliter of BM collected.

### Cell viability & concentration

Cells were diluted, if necessary, in FACSFlow (BD Biosciences). An equal volume of cell suspension and Trypan Blue (Sigma-Aldrich, MO, USA) were mixed and placed in a hemocytometer. Viable and blue-dyed nonviable cells were counted in a microscope. Cellular viability was determined as the viable to total cell ratio and cell concentration as the average viable cells per 1 mm^3^ square × dilution factor × 10^4^.

### MSC cultures

To initiate long-term MSC cultures, MNC were cultured in 175 cm^3^ T-flasks (Falcon™, BD Biosciences) with low glucose DMEM media (Merck, Darmstadt, Deutschland), supplemented with 10% FBS (Biochrome), at 37°C and 5% CO_2_. Cell media was changed weekly and cultures replated when confluence was reached, in either 175 cm^3^ T-flasks or 525 cm^3^ 3-layer flask (Falcon, BD Biosciences). At the first passage, cells were lifted mechanically with a cell scrapper and on subsequent passages, cells were detached with TrypleExpress (Thermo Fisher Scientific), a trypsin-free recombinant enzyme, centrifuged and resuspended in DMEM + 10% FBS.

MSC morphology was confirmed with a reverse microscope and immunophenotyping by flow cytometry.

### Immunophenotyping

A sample of detached MSC was stained with the following fluorochrome conjugated monoclonal antibodies: CD45-FITC, CD34-PE, CD3-PerCP, CD14-PerCP (BD Biosciences), CD44-APC, CD90-APC, CD73- PE (BD Pharmingen, NJ, USA) and CD105-FITC (Bio-Rad, CA, USA).

A 100 μl sample was incubated with the antibodies for 15 min at room temperature in the absence of light. Cells were washed with 2 ml of FACSFlow and centrifuged for 5 min at 1500 r.p.m. After discarding the supernatant, cells were resuspended in 300 μl of FACSFlow and immediately acquired on a FACSCanto II flow cytometer, in accordance with our standard operation procedures. Samples were analysed in duplicate, with a minimum of 50,000 events acquired per tube. The positive region was visually set and also based on 1% of false-positive assumption for negative unstained negative controls [15].

### Adipogenic differentiation

Expanded MSC were tested for adipogenic differentiation assays at different passages. Cells were plated in six-well cell culture plates (Thermo Fisher Scientific) and allowed to adhere. In each plate, four wells were used as negative controls, which were cultured with DMEM + 10% FBS, and two wells were cultured in an adipogenic medium consisting of DMEM + 10% FBS + 50 μM diclofenac+ 175 nM dexamethasone, adapted from existing literature [16], in order to adjust to reagents and drugs available at our institution. Media was changed weekly and plates were observed under the reverse microscope for the development of lipid droplets. Once droplets were observed, their lipid nature was confirmed by staining with oil red O. Before staining, cells were fixated with ethanol, submersed in oil red O (Sigma-Aldrich) twice and the excess dye was washed with distilled water. Plates were immediately observed and photographed under a reverse microscope.

### Mixed lymphocyte reactions

Peripheral blood MNC from two different blood donors was obtained by Lymphoprep gradient centrifugation and resuspended in RPMI 1640 medium, supplemented with 2% FBS and 2% heat-inactivated human AB serum (CSL Behring, PA, USA); stimulator mononuclear cells were irradiated at 20 Gy. Responder mononuclear cells were labelled with the green fluorescent dye CFSE (CellTrace™, Thermo Fisher Scientific), which stains the cytoplasm. Stimulator and responder cells were co-cultured in a 1:1 ratio, in duplicate, in the presence of MSC at 37°C and 5% CO_2_. Positive controls consisted of stimulator and responder cells co-cultured in the absence of MSC in duplicate. Negative controls responder cells and MSC were cultured individually. Proliferation was measured on day 7 by flow cytometry, analyzing the CFSE histogram for the dye dilution, which occurs in each new generation of proliferating cells.

### Product quality control & release criteria

The final cell products underwent established quality control tests, which were planned to adhere to specific criteria, including:

- Cellular viability >80%, assessed by Trypan Blue exclusion method, according to standard procedure assays;
- Sterility of final product performed by direct inoculation of a minimum of 5 ml of pooled culture supernatant in BD BACTEC Plus Aerobic and Anaerobic media (BD Biosciences). Adequacy was reached when all cultures scored negative after a 14-day culture;
- Appropriate phenotype of cultured cells, >90% of CD90, CD105 and CD73 expressing cells and <5% of cells expressing CD34, CD14, CD45 or CD3.

## Results

### MNC recovery & viability

The number of MNC isolated from 1 ml of filtered BM was compared, for each patient, with the MNC recovered from the corresponding collection bag/filter washing. An average of 3.7 × 10^6^ ± 3 MNC was obtained from filtered BM samples, with an average of 1.68 × 10^6^ ± 1.4 MNC recovered, after density gradient centrifuging, from the corresponding 20 bag/filter rinsing procedures. Comparing the volume and number of MNC isolated from each, we can estimate that from rinsing the usually discarded bag/filter, it was possible to obtain as many MNC as from an average 60 ml of filtered BM. In all cases, the recovered MNC had a viability higher than 90%. Cell recovery and viability for each donor are shown in Table 1.

### MSC cultures

The MNC recovered from the collection bag/filter were used to initiate long-term cultures for *ex vivo* expansion of MSC. From these cultures we were able to obtain an average of 17 × 10^6^ MSC, ranging from 8 × 10^6^ to 62 × 10^6^.

### MSC purity

**Figure 1.**
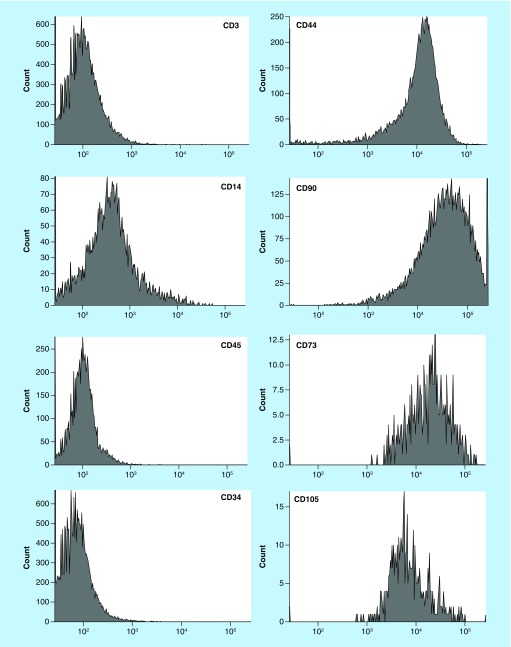
Histograms of the immunophenotype assessed by flow cytometry. Example of one representative culture of mesenchymal stromal cells.

Flow cytometry evaluation for cultured cells immunophenotype was performed either at the time of passage or before cryopreservation. As suggested by the International Society for Cell and Gene Therapy (ISCT), MSC were identified by the surface expression of CD44, CD73, CD90 and CD105 [17]. Cell cultures were considered of adequate purity if more than 90% expressed these markers, with contaminating cells, expressing either of the hematopoietic markers CD34, CD3, CD14 or CD45, being less than 5% (Figure 1). The average number of positive cells (%), for each marker, is shown in Table 2.

**Table 2.** Immunophenotype analysis of mesenchymal stromal cells obtained, for each surface antigen evaluated and standard deviation.

Surface antigen	Average positive cells (%)
CD3	1 ± 0.8
CD14	3 ± 1.9
CD34	0 ± 0.8
CD45	3 ± 1.4
CD44	96 ± 3.6
CD73	95 ± 3.5
CD90	96 ± 3.1
CD105	96 ± 3.5

### Mixed lymphocyte reactions

**Figure 2.**
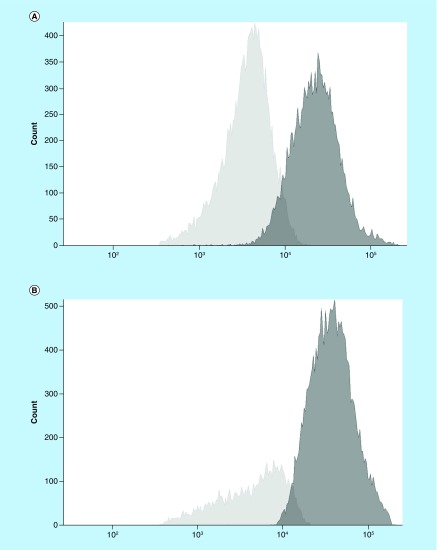
CFSE histograms. Proliferating responder cells are depicted in light gray, in the absence **(A)** and presence **(B)** of mesenchymal stromal cells. Dark gray represents nonproliferating cells.

In four of the long-term MSC cultures, the ability to hamper alloreactivity response was evaluated in a one-way mixed lymphocyte reaction. When comparing the proportion of proliferating responder cells in the absence or presence of MSC, we obtained an average of 64 versus 48%, respectively, which corresponds to a 20% reduction in proliferation (Figure 2). No proliferation was observed in the negative controls.

### Adipogenic differentiation

**Figure 3.**
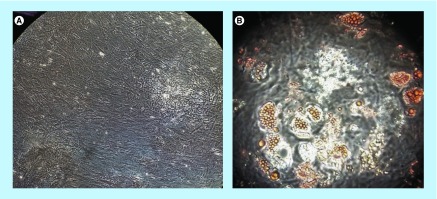
Mesenchymal stromal cell adipogenic differentiation. **(A)** Negative control; **(B)** mesenchymal stromal cell-derived adypocytes with oil red O-stained droplets evident.

We tested the ability of six of our MSC products to differentiate into adipocytes. We adapted existing protocol and defined a simpler media (discussed below). Media was changed twice a week and plates observed until lipid droplets were visible, which occurred after 15–60 days of culture. In all cases, we were able to obtain adipocytes, as shown by the presence of lipid droplets (Figure 3).

## Discussion

A total of 20 BM grafts were harvested with the aim of performing allogeneic HCT. The collection bag and filter system was washed and MNC isolated in order to compare the number of cells obtained with the number of MNC isolated from the actual BM graft. Adjusting each pair of collection bag/filter with the correspondent BM sample, we estimated a MNC recovery equivalent to 60 ml of BM, with a viability higher than 90%. This volume is far superior than most protocols require and most physicians would not be comfortable to collect such a volume from a healthy individual for experimental investigation.

The MNC recovered were then cultured with low glucose DMEM + 10% FBS in order to obtain long-term MSC cultures, which was achieved in all cases. Expanded MSC met the defined quality criteria, with an adequate morphology and immunophenotype (CD105^+^, CD44^+^, CD90^+^, CD73^+^, CD45^-^, CD14^-^ and CD34^-^). To further characterize our MSC, we assessed lineage differentiation by adipogenic differentiation using a novel simpler protocol, shown by oil red staining of MSC-derived adipocytes.

We developed our own protocol for adipogenesis by analysing the pathways involved, already performed by others [16], using the available reagents from our hospital pharmacy and achieved comparable and promising results.

Although, since the first description of trilineage differentiation [18], most groups and the ICST recommend testing for all three lineages to confirm cells as MSC, due to budget constrains we could only test for a single lineage differentiation. However, we are confident that the absence of reports of MSC-like cells presenting the ability to differentiate into only one lineage amends our deficiency. We would therefore recommend further research in this area. Another characteristic of MSC is their ability to downregulate alloreactivity, which we were able to demonstrate *in vitro*, by a consistent 20% reduction of proliferating cells in a one-way mixed lymphocyte reaction. We chose this setup to evaluate the downregulating ability as it mimics the events of GvHD.

It was previously demonstrated that the co-infusion of cells recovered from the filter system, together with the BM graft, may result in lower GvHD and transplant-related mortality. Although MSC expansion was not performed, the authors demonstrated that filter washings contained not only HPC but also stromal progenitors [14]. Previous studies demonstrated that the cell population retained in the collection bag/filter is enriched in stromal progenitors and could be an optimal source for both the isolation of fresh MSC [19,20] or MNC for MSC cultures [21].

We were able to obtain from the MSC expansion an average 17 × 10^6^ MSC, but this number can potentially be increased by protocol optimization, such as supplementing the culture media with platelet lysate rather than FBS and the use of large bioreactors rather than flasks [22–24]. However, it is worth mentioning that patients receiving MSC in a dose lower than predicted demonstrate a good response rate and also, due to the lack of HLA-mediated alloreactivity, it is possible for a patient to receive MSC derived from multiple donors at the same time without side effects or complication [25,26]. More recently, MSC expanded from multiple donors has been reported with great success, demonstrating the low risks of alloreactivity [27,28].

The protocol here described for expansion of MSC has room for optimization, namely with the use of platelet lysate and larger bioreactors, and the full characterization of MSC. Trilineage differentiation was incomplete and proliferation inhibition assays not performed for all samples. In order to be fully compliant with all Good Manufacturing Practices (GMP) required quality controls, endotoxin content and mycoplasma contamination must be performed. Our final product met all previously defined quality criteria (sterility, viability and purity).

## Conclusion

With this study, we demonstrated that after processing for HSCT, the usually discarded BM collection bag/filter system could be a source capable of yielding viable, fully functional and sterile MNC. These cells can be expanded and differentiated into MSC *ex vivo*, maintaining an appropriate phenotype and functional capabilities. By directly comparing with MNC isolated from 1 ml of the actual filtered BM, we were able to calculate 60 ml as the average volume of BM required to equal the number of MNC recovered from the collection bag/filter system.

According to the Center for International Blood and Marrow Transplant Research (CIBMTR) and the European Society Blood and Marrow Transplantation (EBMT), in 2017, over 1000 BM allogeneic HCT were performed in the USA and over 3700 in Europe. Although the recovery of filter-trapped cells was first described over 17 years ago, this resource is still overlooked, or even unknown, by many groups.

Although the MSC obtained were not fully characterized and the expansion protocol described needs further optimization, we strongly believe that MNC recovered from the bag/filter are a suitable alternative to BM for the expansion of MSC, either for investigation or clinical use. The routine recovery of such cells in reference HSCT centers could, in a way similar to a public cord-blood bank, benefit the scientific community.

## Future perspective

There has been an increasing interest in MSC as a therapeutic agent. These cells are easily isolated and expanded *ex vivo*, with diverse clinical indications from immune control (GvHD and autoimmunity) to regenerative medicine (soft tissue). More recently, MSC have been explored as a source of extracellular vesicles, with promising results in cell-free therapies.

We aim to expand MSC from the MNC recovered from the collection bag/filter system with an optimized protocol under GMP conditions and fully characterize the final product, performing trilineage differentiation and proliferation assays, in order to validate this as a source of MSC. The project for the new GMP compliant clean rooms has been approved by the national authority (INFARMED).

We can expect in the next years an increase in the demand for MSC, not only as an off-the-shelf advanced therapy medicinal product (ATMP) but also for translational research, which justify exploring all available sources of MSC.

**Summary points**

- Mesenchymal stromal cells (MSC) are a promising tool for both cellular therapy and regenerative medicine. Their ability to modulate the immune system in a HLA-independent manner makes every MSC donor a universal match.
- Obtaining bone marrow samples with a large enough volume (minimum 20 ml) for *ex vivo* expansion is not always feasible, particularly in investigational settings.
- Before a bone marrow collection can be infused, it must be filtered to remove fat, cell clumps and bone spicules. In the collection bag and filter system are also retained viable cells, which can be recovered.
- From these recovered cells, we isolated the mononuclear fraction by density centrifuging and expanded them into MSC by cell culture in DMEM+FBS.
- To confirm MSC purity we observed the morphology, immunophenotype, immunosuppression and adipogenic differentiation ability.
- We describe here for the first time an alternative adipogenic media, relying on hospital pharmacy-available drugs capable of inducing adipogenesis.
- The systematic recovery of cells from the filter system and expansion of MSC would allow the creation of MSC banks, allowing easy and swift access for research groups.
- Working under GMP conditions, a network of clinical grade MSC providers could function in a manner similar to cord-blood bank, supplying MSC as an off-the-shelf immunotherapy.
